# Catalytic combustion of isopropanol over Co–ZSM-5 zeolite membrane catalysts in structured fixed-bed reactor

**DOI:** 10.1098/rsos.180587

**Published:** 2018-08-29

**Authors:** Xiaotong Zhang, Ying Yan

**Affiliations:** School of Chemistry and Chemical Engineering, Guangdong Provincial Key Lab of Green Chemical Product Technology, South China University of Technology, Guangzhou 510640, People's Republic of China

**Keywords:** structured fixed-bed reactor, cobalt oxides, isopropanol, catalytic combustion, ZSM-5 zeolite membrane

## Abstract

Catalytic combustion of isopropanol in the structured fixed-bed reactor was investigated over Co–ZSM-5 zeolite membrane catalysts. Firstly, ZSM-5 zeolite membrane catalysts with different Si/Al ratios were coated onto the surface of stainless steel fibres via secondary growth method and wet lay-up paper-making method. Then, cobalt oxides were loaded onto the zeolite membranes by impregnation method. The performance of catalytic combustion of isopropanol was conducted over the prepared zeolite membrane catalysts, and the experimental results showed that the catalyst with infinite Si/Al ratio has the highest catalytic activity for the combustion with the lowest *T*_90_ of isopropanol (285°C). Finally, the effects of bed structure, feed concentration, gas hourly space velocity and reaction temperature on the catalytic performance were investigated to analyse the kinetics of isopropanol over the catalyst with infinite Si/Al ratio in the structured fixed-bed reactor. The results showed that the longer residence time could cause higher reaction contact efficiency of isopropanol combustion. *T*_90_ of isopropanol can be dramatically decreased by 105°C in the fixed-bed reactor packed with Co–ZSM-5 zeolite membrane catalysts, compared to the fixed-bed reactor packed with granular catalyst.

## 1. Introduction

Catalytic combustion as the most effective method for the removal of volatile organic compounds (VOCs) from the exhaust gases is widely used in the industrial area. However, from the chemical engineering standpoint, the main problem of VOC removal is the very high dilution of reactants in large gas streams, which implies that the overall process rate is controlled by diffusion [1]. Fixed-bed reactors are widely used in VOC combustion due to their low cost and convenience in operation. Conventional fixed-bed reactors always pack with granular catalysts, which may lead to a higher diffusion resistance according to the particle size of catalysts and voidage of the bed. Therefore, a series of structured catalysts such as honeycomb catalysts (ceramic or metallic) where the active phase is deposited and foam catalysts can overcome these disadvantages. As Fan *et al*. reported, open-cell SiC foams clearly are promising materials for continuous-flow chemical applications such as heterogeneous catalysis and distillation [2,3], the X-ray micro-computed tomography characterization results showed that the spatial voxel size of cellular β-SiC foams was 13.6^3^ µm^3^, and diffusion coefficient of laminar flow within foams was dramatically increased. Xu *et al*. [4] prepared the hydroxyapatite foam as a catalyst for formaldehyde combustion at room temperature based on hydroxyl groups bonded to Ca^2+^ inside channels of HAp which may play an important role in adsorption/activation of HCHO. Ribeiro *et al*. [5] investigated the effects of preparation methods of Pt zeolite coated on cordierite foams on the catalytic performance for toluene combustion. The results showed that the enhanced performances of the structured catalysts for toluene catalytic combustion were due not only to open structure of foams and homogeneous thin zeolite layers deposited on their wall cells but also the fact that the size and location of Pt particles present in zeolite are changed during the dipping steps. In addition, Sebastian *et al*. [6] designed a microreactor with Pt/zeolite catalytic films for the selective oxidation of CO, and the best result can be obtained with total CO combustion temperatures as low as 125°C. However, honeycomb materials that are made of parallel channels have some limitations concerning the laminar flow occurring inside the channels, the mass transfer from the gas phase to the catalytic layer on the walls and difficulties to control temperature for many exothermic or endothermic reactions [7]. Meanwhile, although foam catalysts with open porosity (85–90%) allow increasing the turbulence and the radial mixing [8], the accurate shapes of these foam catalysts should be prepared for the fixed-bed reactor loading due to the mechanical strength. In our previous reports [9–11], a novel gradient paper-like microfibrous zeolite membrane catalyst was prepared for VOC combustion. The results showed that higher contact efficiency with the lower internal and external diffusion resistance was obtained from the prepared catalysts.

The activity of the catalysts should also be adjusted to the enhanced transport properties of the prospective structural reactors in order not to limit the process yield. Noble metals [12–14] and transition metal oxides [15–17] were extensively investigated in the catalytic combustion of VOCs. It is reported that cobalt oxides as the transition metal oxides showed good catalytic activities in VOC combustion [18,19] and have been widely used for the total oxidation of VOCs. A comparison between the catalytic performance of bulk and alumina-supported nanocrystalline cobalt oxide catalysts has been analysed to investigate the influence of crystallite size, nature of the support (*α*, *γ* and mesoporous alumina) and cobalt loading on the catalytic performance of propane combustion in Solsona’s work [20]. Bahlawane prepared monolithic cordierites with low specific surface area, which were uniformly coated with cobalt oxide thin films by chemical vapour deposition method to completely convert methane to CO_2_ below 550°C [21]. Ataloglou *et al*. [22] investigated the effects of the preparation method on the structure–activity of cobalt oxide catalysts supported on alumina for complete benzene oxidation.

Isopropanol as a typical organic pollutant is widely used for solvent and reactant and is released to atmosphere in large concentration. As our extension work, the catalytic performance of isopropanol combustion over cobalt oxides modified zeolite membrane catalysts with different Si/Al ratios will be investigated, and the kinetics of isopropanol in the structured fixed-bed is to be explored by analysing the influence of residence time and reaction temperature on the isopropanol conversion.

## 2. Experimental procedure

### 2.1. Materials

Stainless steel fibres with an average diameter of 6.5 µm and length of 3 mm were purchased from Huitong Advanced Materials Co., Ltd (Hunan, China). Tetrapropylammonium hydroxide (TPAOH, 25% aqueous) was purchased from Quansheng Fine Chemical Co., Ltd (Xi'an, China). Tetraethoxysilane (TEOS, greater than 99%) was purchased from Fuchen Chemical Reagent Factory (Tianjin, China). Ethanol (C_2_H_5_OH, greater than 99.8%), ammonia water (NH_3_, 25–28% aqueous) and sodium aluminate (NaAlO_2_, anhydrous) were all purchased from Sinopharm Chemical Reagent Co., Ltd (Beijing, China). Cobalt nitrate (Co(NO_3_)_2_·6H_2_O) was purchased from Guangzhou Chemical Reagent Factory (Guangzhou, China).

### 2.2. Preparation of Co–ZSM-5 zeolite membrane different Si/Al ratio

First, the paper-like stainless steel fibres were prepared by using wet lay-up paper-making process and modern sintering method according to previous reports of our group [7]. Next, the silicalite-1 seeds were prepared from a mixture solution with the ratio: 9 TPAOH : 25 TEOS : 500 H_2_O : 100 C_2_H_5_OH [23].

Then, ZSM-5 zeolite membranes coating on the surface of stainless steel fibres with different Si/Al ratios (Si/Al = ∞, 180 and 60) were prepared by secondary growth method from a mixture with the mole ratio 100 H_2_O : 0.112 TPAOH : a NaAl_2_O_3_ : 1 TEOS (*a* = 0, 0.008, 0.016). TPAOH solution was mixed with NaAlO_2_ under vigorously stirring for 10 min. Then TEOS was added dropwise into the mixed solution and stirred for 24 h in the magnetic stirrer. The seeded stainless steel fibres were immersed vertically in a Teflon-lined autoclave with the secondary synthesis solution and treated hydrothermally in an oven at 150°C for 48 h. Then, the sample was washed by the deionized water, air-dried (at 100°C for 12 h) and calcined (in air at 550°C for 6 h at a heating rate of 1°C min^−1^) to remove the organic template (TPAOH).

Finally, cobalt oxides modified ZSM-5 zeolite membrane were prepared by impregnation with 30 ml aqueous solution containing known amounts of Co(NO_3_)_2_.6H_2_O of 0.5 M, and the excess of water was removed in an oven at 100°C until dryness for 12 h and subsequently calcined in air for 4 h at the calcination temperature of 500°C.

### 2.3. Characterization

X-ray diffraction (XRD) was performed on a D8 ADVANCE (Bruker, Germany) diffractometer with Cu Kα radiation (40 kV, 40 mA) with 2*θ* range of 1–10° and 5–60° to determine the crystalline phases present in the catalysts. The textural and morphological information of the samples were observed by scanning electron microscopy (SEM, S-3700N, Hitachi, Japan). The samples were coated with Au films before the SEM analysis. Meanwhile, the energy dispersive spectroscopy (Quantax400, Bruker, Germany) for element mapping (EDS mapping) was used to analyse the dispersion of Si, Al, Co and O in the ZSM-5 zeolite membrane. The pore structure of paper-like microfibrous stainless steel was tested by using mercury intrusion porosimetry (MIP), a mercury analyser (AutoPore IV 9500 V1.09, Micromeritics, USA) was used with contact angle of 130.0°, Hg surface tension of 485.0 dyn cm^−1^ and Hg density of 13.6 g ml^−1^. The N_2_ adsorption and desorption isotherms were measured on a 3H-2000PS1 instrument in static measurement mode. All of the samples were outgassed at 200°C for 2 h before measurements. The specific surface area was calculated using the Brunauer–Emmett–Teller (BET) mode, the total pore volume was calculated by the analysis of N_2_ adsorption–desorption isotherms, the micropore volume (Vmicropore) and the mesopore volume (Vmesopore) were calculated by HK (Horvath–Kawazoe) method and BJH (Barrett–Joyner–Halenda) method, respectively.

### 2.4. Kinetics of isopropanol combustion

**Figure 1.**
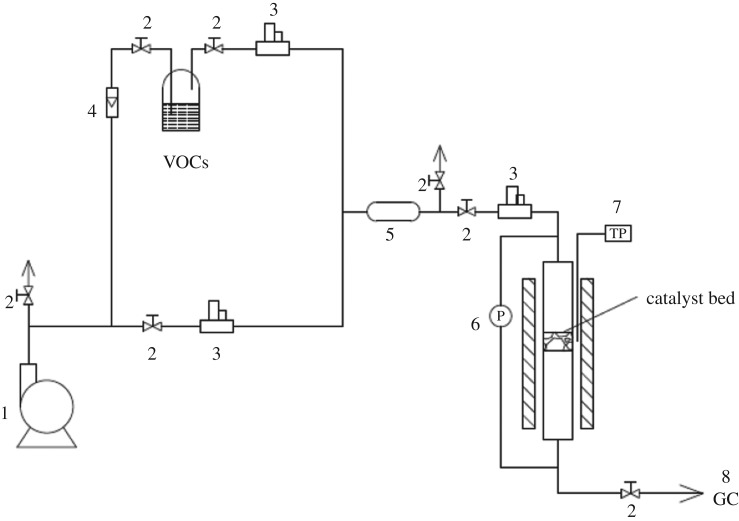
The experimental set-up for isopropanol combustion (1, air compressor; 2, valve; 3, mass flow controller; 4, rotor flow meter; 5, mixing chamber; 6, pressure gauge; 7, temperature probe; 8, gas chromatograph).

**Figure 2.**
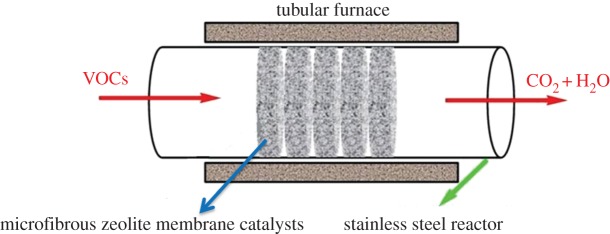
Schematic diagram of the structured fixed-bed reactor.

The catalytic combustion of isopropanol over Co–ZSM-5 zeolite membrane catalysts was carried out in a structured fixed-bed reactor with a stainless steel tube (10 mm i.d., 450 mm length) at atmospheric pressure. The experimental set-up for isopropanol combustion is shown in figure 1 and the schematic diagram of structured fixed-bed reactor is shown in figure 2. The prepared zeolite membrane catalysts were cut into discs with the thickness of 2 mm and placed coaxially in the reactor with a bed height of 1 cm. The temperature in the structured fixed-bed reactor was controlled automatically by E-type thermocouples located in the middle of the bed outside the reactor under temperature-programmed condition. The effect of different bed structure on the catalytic performance was analysed by adjusting the packing ratios of granular Co–ZSM-5 catalyst (*g*) and Co–ZSM-5 zeolite membrane catalysts (*m*). Four types of bed structure were composed of only granular Co–ZSM-5 catalyst, *g* : *m* = 3 : 2, *g* : *m* = 1 : 4, and only Co–ZSM-5 zeolite membrane catalysts, respectively. The isopropanol gas was generated by bubbling air through the saturators, and the accurate flow rate was adjusted by mass flow controllers. The reaction temperature was adjusted from 140 to 320°C (catalytic combustion temperature) with a heating rate of 5°C min^−1^, the reaction temperature was measured every 20°C which kept for 30 min at the desired temperature. The reactants and the products of the reaction were analysed offline by using GC (Agilent 7890A, Palo Alto, CA, USA) equipped with an FID detector.

## 3. Results and discussion

### 3.1. X-ray diffraction

**Figure 3.**
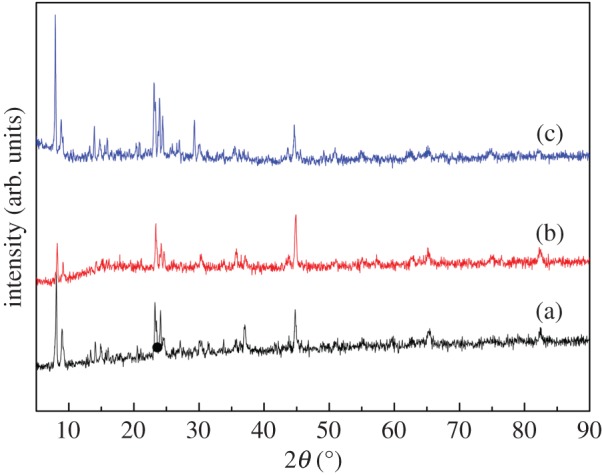
XRD patterns of samples: (a) Co–ZSM-5 (Si/Al = ∞); (b) Co–ZSM-5 (Si/Al = 60); (c) Co–ZSM-5 (Si/Al = 180).

The XRD patterns of Co–ZSM-5 zeolite membrane catalysts coated on microfibrous stainless steel with different Si/Al ratios (60, 180, ∞) are shown in figure 3. As can be seen from figure 3, two diffraction peaks appearing at 2*θ* = 7–9° and 23–25° in all the samples suggest the standard phase of ZSM-5 zeolite [24]. Meanwhile, the diffraction peaks appearing at 2*θ* = 43–55° in all the samples can be attributed to the characteristic peak of micro stainless steel fibre, as in our previous report [7]. For cobalt oxides dispersion, all the samples give the same diffraction peaks in the ranges of 2*θ* = 36.5°, 45°, 52.6° and 65.7°, which matches well with the diffraction patterns of crystalline cubic Co_3_O_4_ (JSPDS: 42–1467). XRD patterns of Co–ZSM-5 zeolite membranes with different Si/Al ratios are very similar and no specific orientation or orientation differences between the samples could be derived from these patterns, which has been confirmed in other literature as well [25,26].

### 3.2. Scanning electron microscopy

**Figure 4.**
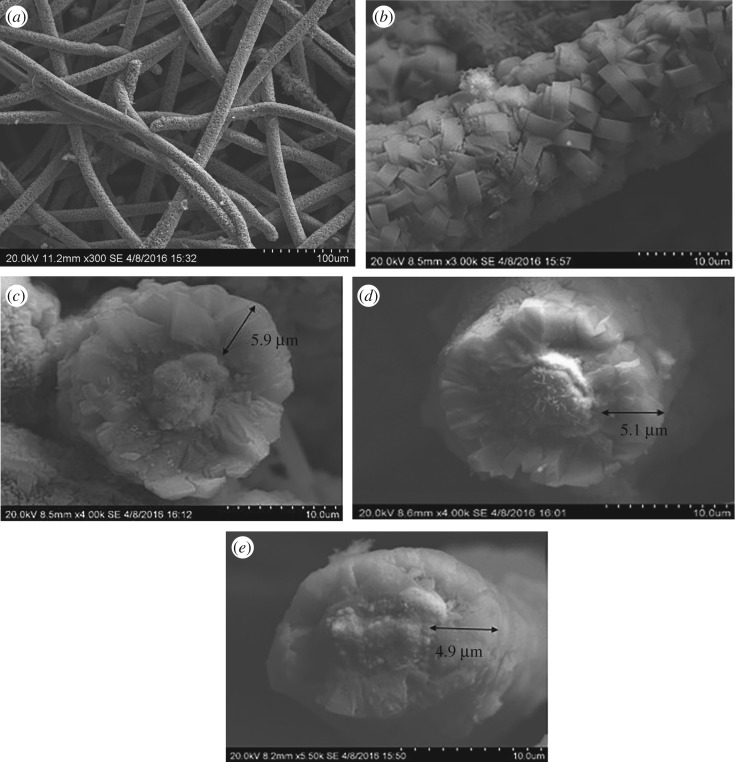
SEM images of samples: (*a*) ZSM-5 zeolite membrane; (*b*) Co–ZSM-5 (Si/Al = ∞); (*c*) cross-section of C-/ZSM-5 (Si/Al = ∞); (*d*) cross-section of Co–ZSM-5 (Si/Al = 180); (*e*) cross-section of Co–ZSM-5 (Si/Al = 60).

Morphology and structure of the synthesized Co–ZSM-5 zeolite membrane catalysts coated on microfibrous stainless steel with different Si/Al ratios (60, 180, ∞) were analysed by SEM, and the results are shown in figure 4. The prepared Co–ZSM-5 zeolite membrane catalysts coated on microfibrous stainless steel indicates a three-dimensional network structure as shown in figure 4*a*, and the typical coffin-shaped crystals of ZSM-5 were obtained on the surface of micro stainless steel fibres in figure 4*b*. ZSM-5 zeolite membrane grows well on the surface of the PSSF supports, with a continuous dispersion and random orientation. As the Si/Al ratio increased, the thickness of the zeolite membrane was increased from 4.9 µm in figure 4*d* to 5.9 µm in figure 4*c*. According to Hensen's report [25], at high Al content, the presence of ZSM-5 zeolite membrane crystals is coffin-shaped, and these coffin shapes are better intergrown with increasing Si/Al ratio, which lead to a continuous thicker membranes.

**Figure 5.**
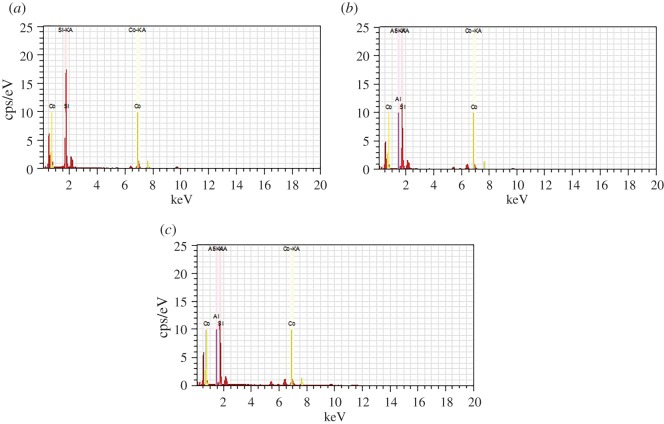
EDS elemental analysis spectra of samples: (*a*) Co–ZSM-5 (Si/Al = ∞); (*b*) Co–ZSM-5 (Si/Al = 180); (*c*) Co–ZSM-5 (Si/Al = 60).

**Figure 6.**
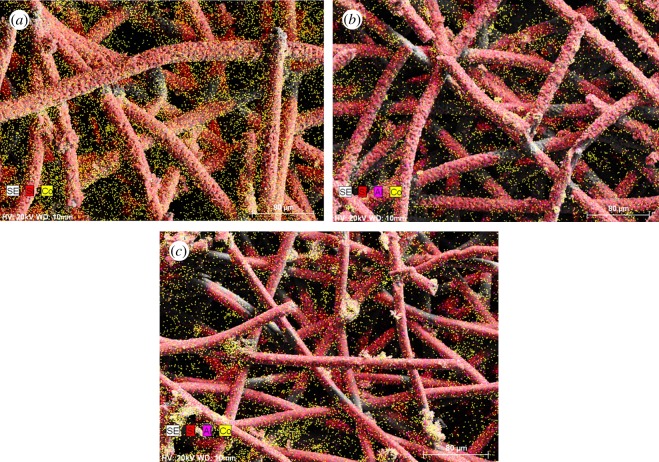
EDS elemental mapping of samples: (*a*) Co–ZSM-5 (Si/Al = ∞); (*b*) Co–ZSM-5 (Si/Al = 180); (*c*) Co–ZSM-5 (Si/Al = 60).

According to the EDS results in figures 5 and 6, the content of Al was decreased as the Si/Al ratio increased. The Co element in yellow colour covered the most part of the Co–ZSM-5 zeolite membrane catalyst with Si/Al = ∞ and its distribution is particularly intensive and uniform. However, as the content of Al increased, cobalt oxides did not disperse uniformly over ZSM-5 zeolite membrane; obviously, agglomeration of the cobalt oxides may be found in the EDS mapping results.

### 3.3. N_2_ adsorption/desorption isotherms analysis

**Figure 7.**
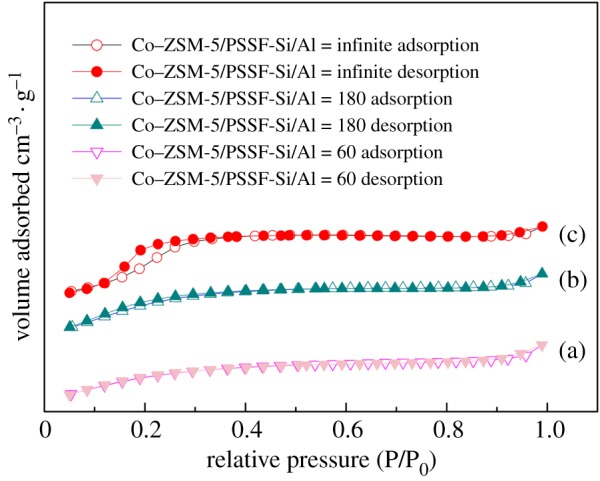
Nitrogen adsorption/desorption isotherms of Co–ZSM-5 zeolite membrane catalyst at 77 K.

The pore structure of paper-like microfibrous stainless steel was tested by using MIP [27], and the results are shown in table 1. It is clear to see that the prepared paper-like microfibrous stainless steel without ZSM-5 zeolite membrane coating was mainly composed of macropores with the average pore diameter of 45.6 nm, and a higher porosity structure was obtained with the porosity of 98.7%, which is much higher than that of the foam materials (85–90%) [8]. Therefore, the higher porosity structure can effectively reduce the external diffusion resistance of the fluid, and the internal diffusion resistance can be dramatically decreased based on the micropore structures in micron thickness of ZSM-5 zeolite membrane coated on the microfibrous stainless steel. The results of the specific surface areas and porosity properties based on the N_2_ adsorption/desorption isotherms are shown in figure 7 and table 2. The N_2_ adsorption/desorption isotherms of all the samples fitted for a typical microporous feature of such material (type I adsorption isotherm) [28]. From the BET results, the specific surface areas increase with increasing the Si/Al molar ratios of the samples. This result matches well with the result of EDS mapping; agglomeration of the cobalt oxides may block the pores and significantly reduce the specific surface area of the catalyst. Similar results also can be obtained in Shirazi's work [29].

**Table 1.** The pore structures of the paper-like microfibrous stainless steel.

property	microfibrous
total intrusion volume (ml g^−1^)	3.7
total pore area (m^2^ g^−1^)	0.3
median pore diameter (volume) (nm)	66519.3
median pore diameter (area) (nm)	52589.2
average pore diameter (4 V/A) (nm)	45570.3
bulk density at 0.51 psia (g ml^−1^)	0.3
apparent (skeletal) density (g ml^−1^)	20.3
porosity (%)	98.7

**Table 2.** Pore structure characteristics of samples.

sample	micropore volume (cm^−3^ g^−1^)	mesopore volume (cm^−3^ g^−1^)	total volume (cm^−3^ g^−1^)	BET surface area *S*_BET_ (m^2^ g^−1^)
Co–ZSM-5 (Si/Al = 60)	0.04	0.02	0.07	115
Co–ZSM-5 (Si/Al = 180)	0.05	0.03	0.09	160
Co–ZSM-5 (Si/Al = ∞)	0.05	0.04	0.10	192

### 3.4. Kinetics of isopropanol combustion

#### 3.4.1. Effect of Si/Al ratio

**Figure 8.**
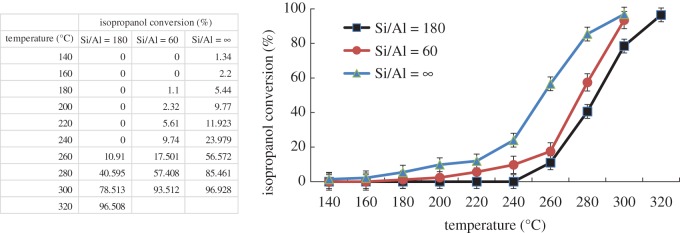
Catalytic performances for isopropanol combustion over Co–ZSM-5 zeolite membrane catalysts with different Si/Al ratio (4.5 mg l^−1^ of isopropanol in the feed gas and GHSV of 15 286 h^−1^).

Catalytic performance of isopropanol combustion over Co–ZSM-5 zeolite membrane catalysts coated on microfibrous stainless steel with different Si/Al ratios (60, 180, ∞) under feed concentration of 4.5 mg l^−1^ and a constant gas flow rate (gas hourly space velocity (GHSV) of 15 286 h^−1^) is shown in figure 8. The 50% and 90% conversion temperatures (denoted as *T*_50_ and *T*_90_) for isopropanol combustion can be obtained from the curves. As can be seen, Co–ZSM-5 zeolite membrane catalysts with Si/Al = ∞ show the best catalytic performance with *T*_50_ of 256°C and *T*_90_ of 285°C, and the activity order of prepared catalysts is as follows: Co–ZSM-5 (Si/Al=∞) > Co–ZSM-5 (Si/Al = 60) > Co–ZSM-5 (Si/Al = 180).

#### 3.4.2. Effect of bed structure

**Figure 9.**
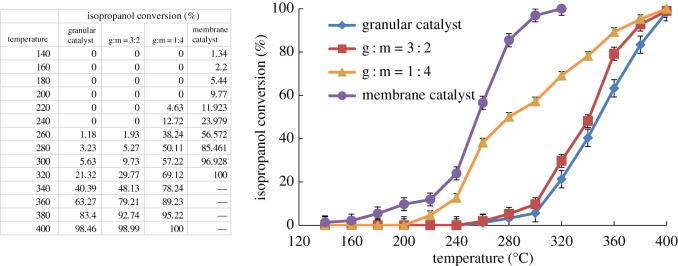
Catalytic performances of isopropanol combustion in different bed structure (4.5 mg l^−1^ of isopropanol in the feed gas and GHSV of 15 286 h^−1^).

Catalytic performances of isopropanol combustion in structured fixed-bed reactors packed with different ratios of Co–ZSM-5 zeolite membrane catalysts with Si/Al = ∞ and granular Co–ZSM-5 zeolite catalyst under feed concentration of 4.5 mg l^−1^ and a constant gas flow rate (GHSV of 15 286 h^−1^) is shown in figure 9. As the zeolite membrane catalysts packing ratio in the structured fixed-bed reactor increased, *T*_50_ and *T*_90_ of isopropanol conversion decreased. Compared to the fixed-bed reactor packed with only granular catalyst, *T*_50_ of isopropanol conversion can be decreased by 95°C and *T*_90_ can be decreased by 105°C in the fixed-bed reactor packed with only Co–ZSM-5 zeolite membrane catalysts. The cobalt oxides dispersed well onto the ZSM-5 zeolite membrane due to the higher porosity of the microfibrous support and open structure of zeolite membrane. As mentioned, the mass transfer enhancement can be attributed to the reduction in external and internal diffusion based on the prepared zeolite membrane catalyst, which can effectively improve the contact efficiency, and the reaction temperature can be dramatically decreased.

#### 3.4.3. Effect of feed concentration and gas hourly space velocity

**Figure 10.**
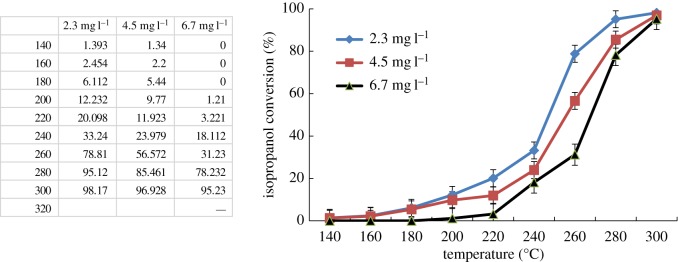
Catalytic performances for isopropanol combustion over Co–ZSM-5 (Si/Al = ∞) with different feed concentration (GHSV of 15 286 h^−1^).

Firstly, the behaviours of catalytic oxidation of isopropanol were investigated by recording the conversion profiles at different feed concentrations. The experiment was carried out by using different feed concentrations (2.3–6.7 mg l^−1^) with a constant gas flow rate (GHSV of 15 286 h^−1^), the results are shown in figure 10. The values of *T*_50_ and *T*_90_ of isopropanol conversion increased slightly as the feed concentration increased. When the feed concentration increased, isopropanol molecules that adsorbed on the active sites are in competition with oxygen, at a high feed concentration. More isopropanol molecules are adsorbed and the surface oxygen is smaller and becomes the controlling factor, and consequently, the conversion of isopropanol is lower [30].

**Figure 11.**
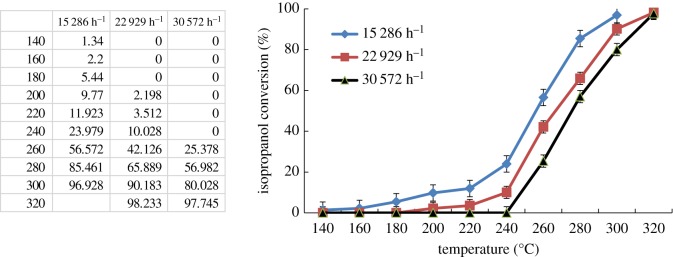
Catalytic performances for isopropanol combustion over Co–ZSM-5 (Si/Al = ∞) with different GHSV (4.5 mg l^−1^ of isopropanol in the feed gas).

Meanwhile, GHSV plays a very important role in the catalytic combustion because the resistance time is relevant to GHSV. The catalytic performance for isopropanol combustion in the fixed-bed reactor was investigated by using different GHSV (15 286–30 572 h^−1^) with a constant inlet concentration of isopropanol in the feed stream (4.5 mg l^−1^). The experimental results in figure 11 showed that the values of *T*_50_ of isopropanol conversion increased slightly from 256 to 272°C and *T*_90_ increased from 285 to 308°C, as the space velocity increased from 15 286 to 30 572 h^−1^. As the gas flow rate increased, the residence time of isopropanol molecules decreased in the fixed-bed reactor, which indicated the contact time of isopropanol molecules passing through the fixed-bed reactor over catalysts was also decreased, according to Choudhary's report [31].

#### 3.4.4. Effect of reaction temperature

**Figure 12.**
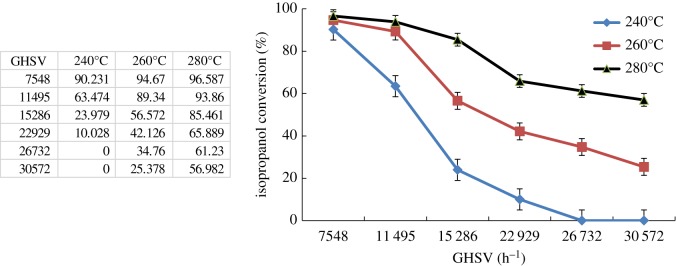
Catalytic performances for isopropanol combustion over Co–ZSM-5 (Si/Al = ∞) at different reaction temperature (feed concentration of 4.5 mg l^−1^).

The effect of reaction temperature (240, 260 and 280°C) on the catalytic performance of isopropanol combustion in the fixed-bed reactor was conducted under the feed concentration of 4.5 mg l^−1^ with different GHSV. As can be seen in figure 12, as the reaction temperature increased, the isopropanol conversion increased dramatically. However, the isopropanol conversion curve at the reaction temperature of 240°C is much sharper than that at the reaction temperature of 280°C. In other words, the influence of residence time on isopropanol conversion can be reduced under higher reaction temperature. The possible reason may be that the reaction rate of isopropanol combustion at higher temperature is much faster. As the GHSV increased, the decrease in the contact time of isopropanol molecules had little influence on the catalytic performance of isopropanol combustion.

## 4. Conclusion

Co–ZSM-5 zeolite membrane catalysts with different Si/Al ratios were prepared for isopropanol combustion in the structured fixed-bed reactor. The catalyst with infinite Si/Al ratio has the best catalytic activity due to the better specific surface area and better dispersion of cobalt oxides. *T*_90_ of isopropanol in the structured fixed-bed reactor packed only with membrane catalyst showed the best catalytic performance, the *T*_90_ of isopropanol combustion can be decreased by nearly 100°C compared to the structured fixed-bed reactor packed only with granular catalyst. According to the kinetics of isopropanol over Co–ZSM-5 zeolite membrane catalysts (Si/Al = ∞), *T*_90_ and *T*_50_ of isopropanol combustion decreased dramatically as the feed concentration and GHSV decreased. In addition, the influence of residence time on the catalytic performance can be reduced when the reaction temperature increased.

## Data accessibility

This article does not contain any additional data.
